# Five Italian Families with Two Mutations in *BRCA* Genes

**DOI:** 10.3390/genes11121451

**Published:** 2020-12-03

**Authors:** Maria Teresa Vietri, Gemma Caliendo, Giovanna D’Elia, Marianna Resse, Amelia Casamassimi, Pellegrino Biagio Minucci, Concetta Dello Ioio, Michele Cioffi, Anna Maria Molinari

**Affiliations:** 1 Department of Precision Medicine, University of Campania “Luigi Vanvitelli”, Via L. De Crecchio, 80138 Naples, Italy; marianna.resse@unicampania.it (M.R.); amelia.casamassimi@unicampania.it (A.C.); pellegrinob.minucci@unicampania.it (P.B.M.); michele.cioffi@unicampania.it (M.C.); annamaria.molinari@unicampania.it (A.M.M.); 2 Unity of Clinical and Molecular Pathology, University of Campania “Luigi Vanvitelli”, 80138 Naples, Italy; gemma.caliendo@unicampania.it (G.C.); giovanna.delia@policliniconapoli.it (G.D.); 3 Oncology Unit, Hospital “Andrea Tortora,” ASL Salerno, 84016 Pagani, Italy; c.delloioio@aslsalerno.it

**Keywords:** double heterozygosity (DH), double mutations (DM), *BRCA1*, *BRCA2*, hereditary breast and ovarian cancer

## Abstract

Double heterozygosity (DH) in *BRCA1* and *BRCA2* genes and double mutation (DM) in *BRCA1* or *BRCA2* are extremely rare events in the general population, and few cases have been reported worldwide so far. Here, we describe five probands, all women, with breast and/or ovarian cancer and their families. Particularly, we identified two probands with DH in the *BRCA1/2* genes with a frequency of 0.3% and three probands with DM in the *BRCA2* gene with a frequency of 0.5%. The DH *BRCA1* c.547+2T>A (IVS8+2T>A)/*BRCA2* c.2830A>T (p.Lys944Ter) and *BRCA1* c.3752_3755GTCT (p.Ser1253fs)/*BRCA2* c.425+2T>C (IVS4+2T>C) have not been described together so far. The DM in *BRCA2*, c.631G>A (p.Val211Ile) and c.7008-2A>T (IVS13-2A>T), found in three unrelated probands, was previously reported in further unrelated patients. Due to its peculiarity, it is likely that both pathogenic variants descend from a common ancestor and, therefore, are founder mutations. Interestingly, analyzing the tumor types occurring in DH and DM families, we observed ovarian cancer only in DH families, probably due to the presence in DH patients of *BRCA1* pathogenic variants, which predispose one more to ovarian cancer onset. Furthermore, male breast cancer and pancreatic cancer ensued in families with DM but not with DH. These data confirm that *BRCA2* pathogenic variants have greater penetrance to develop breast cancer in men and are associated with an increased risk of pancreatic cancer.

## 1. Introduction

The presence of genetic factors plays a large role in breast cancer (BC) and ovarian cancer (OC) onset [1,2]. About 5–10% of breast tumors and 10–15% of ovarian tumors are hereditary, and approximately 30% of cases are attributed to pathogenic variants in *BRCA1/2* genes [3]. Many nongenetic factors, such as the degree of economic development, social status, and lifestyle factors (such as obesity) affect the probability of developing BC or OC [4], and the risks associated with *BRCA1* and *BRCA2* pathogenic variants differ in various geographical areas, highlighting the importance of evaluating the risk for each patient regarding their own genetic and environmental context [5].

The characterization of germline pathogenic variants in the *BRCA1* and *BRCA2* genes are relevant for prevention setting and for the clinical management of hereditary breast and ovarian cancer (HBOC) [6].

The mutation frequency of these genes occurs within the population in a variable way as to 1/400 up to 1/800 individuals, and the prevalence of founder mutations in some ethnic groups is much higher, as in Ashkenazi Jewish, where it is 1/40 [7]. Double heterozygosity (DH) of *BRCA1* and *BRCA2* is extremely a rare event in general population [7]. It is estimated at 1.8% in Ashkenazi Jewish, due to founder mutations, whereas in non-Ashkenazi Jewish it is in the range of 0.22–0.83% [8]. Indeed, few DH cases have been reported worldwide [9,10,11,12,13,14,15,16,17,18,19,20,21,22,23,24,25,26,27,28,29,30,31] as well as in the Italian population [7,32,33,34,35,36]. Additionally, the DM in the same gene, *BRCA1* or *BRCA2*, is rare and reported in few cases [23,24,36,37,38,39,40].

Here, we describe two cases of DH in *BRCA1*/*BRCA2* genes and three cases of DM in the *BRCA2* gene in five probands with breast and ovarian cancer and in their families.

## 2. Materials and Methods

### 2.1. Patients

This study was carried out in accordance with the World Medical Association Helsinki Declaration, adopted in 1964 and amended in 1975, 1983, 1989, 1996, and 2000. Informed consent was obtained from all subjects, and the study was approved and conducted according to the ethical guidelines of the University of Campania “Luigi Vanvitelli” (n.469-23 July 2019).

The five DH cases described were ascertained among 645 patients tested for *BRCA1* and *BRCA2* pathogenic variants at our center, Unity of Clinical and Molecular Pathology, University of Campania “Luigi Vanvitelli” of Naples. All the selected patients received genetic counselling. The case history was collected and the pedigree was generated with (i) the personal history of cancers (ii) the family history of breast/ovarian and other cancers.

Out of 5 proband, all women, three were affected with BC, one bilateral breast cancer (bBC) and one ovarian cancer (OC). All came from the same region, Campania, a region of Southern Italy with Caucasian ethnicity. Mutational analysis was extended to family members of mutated probands, and at least one family member was available for genetic testing.

### 2.2. Mutation Analysis

Peripheral blood samples were collected from patients. The extraction of genomic DNA from peripheral blood lymphocytes was performed using the Wizard Genomic DNA purification kit (Promega Corporation, Madison, WI, USA). A mutational analysis of exons and adjacent intronic regions of *BRCA1/BRCA2* genes was performed with next-generation sequencing (NGS), as previously described [41]. The presence of the pathogenic variant was confirmed on a second blood sample by Sanger sequencing, as previously described [42].

## 3. Results

We observed two cases of DH (0.3%) in *BRCA1/2* genes in a cohort of 645 probands affected with breast or ovarian cancer. Particularly, we found one patient with DH in a cohort of 533 (0.2%) BC patients and one patient with DH in a cohort of 59 (1.7%) OC patients. In addition, we found three cases of DM (0.5%) in the *BRCA2* gene.

The clinical characteristics of the probands and the corresponding pathogenic variants are listed in Table 1 and Table 2.

All the pathogenic variants had been previously described as “Pathogenic” in the public variant databases ClinVar (https://www.ncbi.nlm.nih.gov/clinvar/variation/) [43], except the *BRCA2* c.425+2T>A (IVS4+2T>C), classified as “Likely Pathogenic”.

### 3.1. Family 1

**Figure 1:**
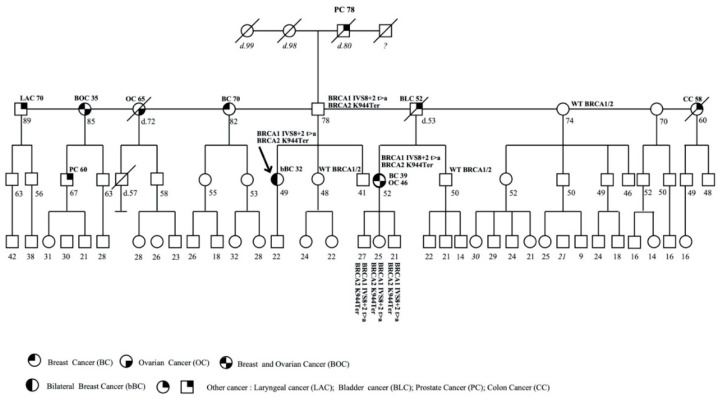
Pedigree of family 1 carrying the double heterozygosity (DH) in *BRCA1* c.547+2T>A (IVS8+2T>A) and *BRCA2* c.2830A>T (p.Lys944Ter). The ages at diagnosis are indicated in brackets.

The patient with pathogenic variants c.547+2T>A (IVS8+2T>A) in *BRCA1* and c.2830A>T (p.Lys944Ter) in *BRCA2* has already been described [35]. After 2013, the mutation analysis was extended to other family members. The proband was a 49-year-old woman affected with bilateral BC. The patient referred other cases of tumors in her family (Figure 1).

The mother’s family is not displayed in the pedigree because no cancer types were reported from the maternal side. It is noteworthy that mutational analysis showed that the proband’s mother did not carry either of the pathogenic variants, while the unaffected father carried both the *BRCA1* and *BRCA2* pathogenic variants. The analysis was performed in the unaffected sister of 48 years, one healthy paternal aunt of 74 years, and one paternal cousin affected with BC and OC, diagnosed at 39 and 46 years, respectively. DH was carried by her cousin, affected with BC and OC, while the paternal aunt and the sister of proband reported a normal profiling of both *BRCA1* and *BRCA2*.

Subsequently, molecular analysis was extended to three sons of the cousin with DH, two men of 27 and 21 years, respectively, and one 25-year-old woman, and to the brother of 50 years; all individuals were healthy. DH was carried by all sons, while the brother carried a wild-type profiling of both genes.

### 3.2. Family 2

The proband was a 36-year-old woman affected with OC. Mutational analysis revealed the *BRCA1* and *BRCA2* pathogenic variants, c.3752_3755GTCT (p.Ser1253fs) and c.425+2T>C (IVS4+2T>C), respectively.

**Figure 2:**
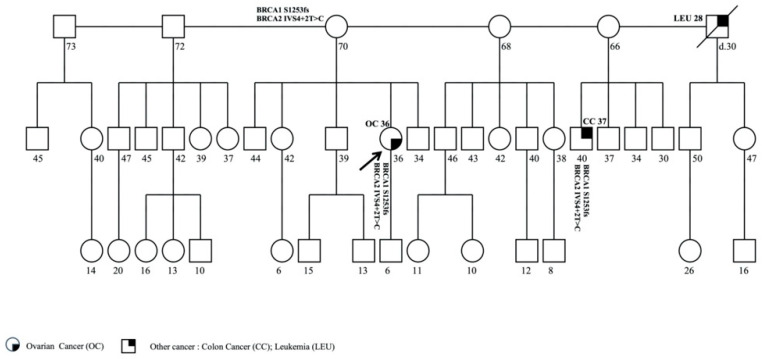
Pedigree of family 2 carrying the DH in *BRCA1* c.3752_3755GTCT (p.Ser1253fs) and *BRCA2* c.425+2T>C (IVS4+2T>C). The ages at diagnosis are indicated in brackets.

The proband reported other cases of cancer in family on the maternal side (Figure 2). The mother’s brother, who died at 30 years, was affected with leukemia and diagnosed at 28 years, while the cousin of 40 years was affected with colon cancer (CC), diagnosed at 37 years. The analysis was performed in the unaffected mother, who was 70 years old, and in the cousin affected with CC; both subjects showed DH. The proband’s son was not tested because he was 6 years old. To date, no other family member has consented to the testing.

### 3.3. Family 3

The proband was a 46-year-old woman affected with BC, diagnosed at 45 years. Molecular analysis showed two pathogenic variants in *BRCA2* gene, c.631G>A (p.Val211Ile) and c.7008-2A>T (IVS13-2A>T).

**Figure 3:**
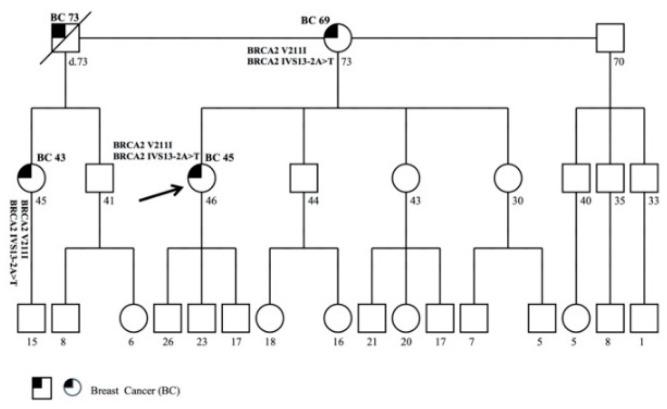
Pedigree of family 3 carrying the DM in *BRCA2* c.631G>A (p.Val211Ile) and c.7008-2A>T (IVS13-2A>T). The ages at diagnosis are indicated in brackets.

The patient reported other cases of BC in her family on the maternal side (Figure 3). The mother was affected with BC, diagnosed at 69 years; the mother’s brother, affected with male BC, died at 73 years a few months after diagnosis. The latter’s daughter, who was 45 years old, was affected with BC and diagnosed at 43 years. Molecular analysis, performed in the mother and in the proband’s cousin of 45 years, revealed DM in both family members. To date, no other family member has received genetic testing.

### 3.4. Family 4

The proband was a 77-year-old woman affected with BC who was diagnosed at 65 years. She reported two pathogenic variants in the *BRCA2* gene, c.631G>A (p.Val211Ile) and c.7008-2A>T (IVS13-2A>T).

**Figure 4:**
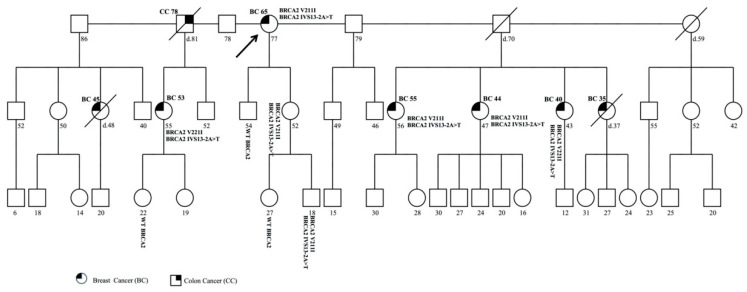
Pedigree of family 4 carrying the DM in *BRCA2* c.631G>A (p.Val211Ile) and c.7008-2A>T (IVS13-2A>T). The ages at diagnosis are indicated in brackets.

She showed a further seven BC cases in the family and one case of CC (Figure 4). Molecular analysis was performed in the unaffected children of 52 and 54 years old, with the son of 54 years reporting a normal *BRCA2* profile and the daughter of 52 years displaying DM. The analysis was also extended to the latter’s sons, of 27 and 18 years, and this revealed the presence of DM only in the son of 18 years.

In an analysis performed in the proband’s nieces, four women of 55, 56, 47, and 43 years, all affected with BC, showed DM in all of them.

Subsequently, one of the daughters of the 55-year-old niece with BC and DM was tested, with a negative result. To date, the other family members have decided not to undergo genetic testing.

### 3.5. Family 5

The proband was a 62-year-old woman affected with BC who was diagnosed at 60 years. Molecular analysis showed two pathogenic variants in *BRCA2* gene, c.631G>A (p.Val211Ile) and c.7008-2A>T (IVS13-2A>T).

**Figure 5:**
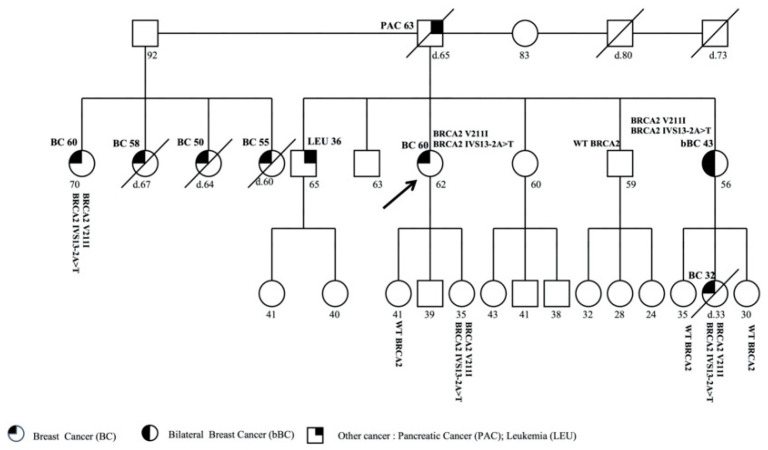
Pedigree of family 5 carrying the DM in *BRCA2* c.631G>A (p.Val211Ile) and c.7008-2A>T (IVS13-2A>T). The ages at diagnosis are indicated in brackets.

The patient reported other cancer cases of in her family (Figure 5); a sister was affected with bBC and was diagnosed at 43 years, and the brother, who was 65 years old, received a diagnosis of leukemia at 36 years. A 70-year-old paternal cousin was affected with BC and diagnosed at 60 years. Moreover, the proband’s father, who was affected with pancreatic cancer, died at 65 years, and three paternal cousins with BC died at 67, 60, and 64 years.

The analysis was performed in the unaffected daughters of 35 and 41 years. DM was identified only in the daughter of 35 years. Moreover, the mutational analysis was executed in the sister of 56 years with bBC, who showed DM in the *BRCA2* gene. Subsequently, the analysis was extended to the unaffected daughters of the latter, of 35 and 30 years, who had negative results, and the daughter with BC, diagnosed at 32 years, who showed the DM and died at the same age, a few months after testing. In addition, the analysis was extended to the proband unaffected brother of 59 years old and reported a wild-type profiling, and to the proband’s cousin of 70 years who was affected with BC, reporting DM. The presence of DM in the paternal cousin indicates that the *BRCA2* double mutation was very likely inherited from the paternal side.

Table 3 summarizes the results of the genetic tests carried out on probands with DH and family members of the probands undergoing the test. No family member has inherited a single pathogenic variant. All the family members tested, affected with cancer, showed DH. Out of nine subjects with DH, four were affected with cancer (2 probands + 2 family members) and 1/4 (25%) was affected with bilateral breast cancer (bBC), 1/4 (25%) with OC, 1/4 (25%) with BC and OC, and 1/4 (25%) with CC, while five were healthy. The phenotypic expression in DH subjects varied from bBC at the age of 32 to healthy at the age of 78.

Table 4 summarizes the results of the genetic tests carried out on probands with DM and family members of the probands undergoing the test. No family member has inherited a single pathogenic variant. All the family members tested who were affected with cancer showed DM. Out of 15 subjects with DM, 12 were affected with cancer (3 probands + 9 family members), 11/12 (92%) were affected with BC and 1/12 (8%) with bBC, while three were healthy. The phenotypic expression in DM subjects varied from BC at the age of 32 to healthy at the age of 52.

**Figure 6:**
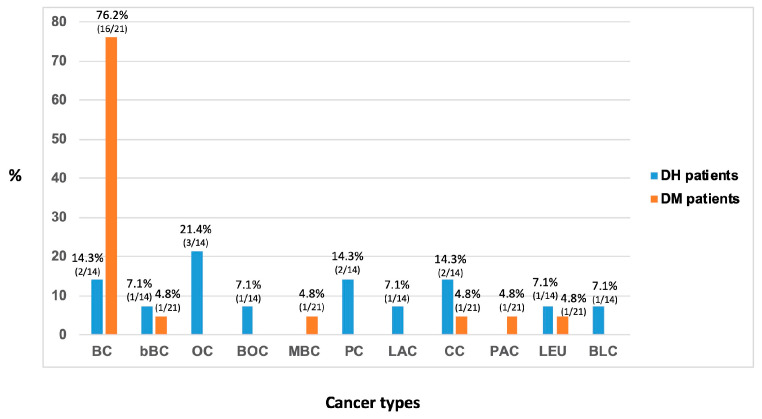
Cancer types occurring in families with DH and DM. BC: breast cancer; bBC: bilateral breast cancer; OC: ovarian cancer; BOC: breast ovarian cancer; MBC: male breast cancer; PC: prostate cancer; LAC: laryngeal cancer; CC: colon cancer; PAC: pancreatic cancer; LEU: leukemia; BLC: bladder cancer.

Figure 6 reports the number and percentages of various types of tumors occurring in families with DH in *BRCA1/2* and DM in *BRCA2*.

The average age of onset of various cancer types occurring in families with DH in *BRCA1/2* and DM in *BRCA2* is reported in Table 5.

Additionally, we completed a review of the worldwide literature using the PubMed database (https://www.ncbi.nlm.nih.gov/pubmed). From 1997 to 2019, 31 articles described a DH in *BRCA1/2* and 7 articles described a DM in *BRCA2*. The results are summarized in Table 6 and Table 7, respectively.

## 4. Discussion

In the last few years, the development of next-generation sequencing (NGS) has enhanced the ability to test for many genes simultaneously, has allowed progress in the field of cancer genetics and lowered the cost of genetic testing. This advance has led to greater insights into hereditary cancer and consequently to confer significant risk for either breast or ovarian cancer [3].

To date, the co-existence in an individual of DH in *BRCA1* and *BRCA2* genes is a very rare condition in most populations and, therefore, little is known about the pathological characteristics of tumors in patients with DH and their family history. Indeed, only 31 studies worldwide have reported patients with DH in the *BRCA1/2* genes. Table 6 is a list of DH cases identified in Ashkenazi Jewish population and non-Ashkenazi [7,9,10,11,12,13,14,15,16,17,18,19,20,21,22,23,24,25,26,27,28,29,30,31,32,33,34,35,36].

In this study we have identified two cases with DH for pathogenic variants in *BRCA1/2* genes with a frequency of 0.3%, which falls within the range previously reported (0.3–1.8%) [44,45]. Indeed, in different ethnic groups with BC and/or OC, a value of 0.3% DH was reported [44], while in the Ashkenazi population a percentage of 1.8% was found [8].

To our knowledge, the DH found in family 1 *BRCA1* c.547+2T>A (IVS8+2T>A)/*BRCA2* c.2830A>T (Lys944Ter) and DH found in family 2, *BRCA1* c.3752_3755GTCT (p.Ser1253fs)/*BRCA2* c.425+2T>C (IVS4+2T>C) (see Table 1), have not been described together so far. The data obtained from the analysis on families show that these pathogenic variants co-segregate. The *BRCA1* c.3752_3755GTCT (p.Ser1253fs) was previously observed in women of Italian origin with OC [46]. Moreover, the *BRCA2* c.425+2T>C (IVS4+2T>C), occurring in family 2, has not been reported in individuals with *BRCA2*-related disease and in association with other *BRCA* mutations. This variant may disrupt the consensus splice site and lead to a loss of protein function. The currently available evidence indicates that additional data are needed to prove that this variant is pathogenic [47]. Additionally, it could represent the first case in which this variant is found in a patient with OC and associated with another *BRCA* pathogenic variant.

Moreover, we have identified three unrelated cases with the same DM in *BRCA2* gene with a frequency of 0.5%. To our knowledge, very few studies worldwide have reported patients with DM in *BRCA2* gene (Table 7).

The DM in *BRCA2*, c.631G>A (p.Val211Ile)/c.7008-2A>T (IVS13-2A>T), as shown in Table 7, was previously reported in Italian unrelated patients [36,37,38,39]. Moreover, c.631G>A (p.Val211Ile) was firstly reported as a single mutation in an Italian breast and ovarian cancer patient [48].

Both mutations alter normal mRNA splicing, leading to the expression of a truncated protein [37,39]. Since germinal mutations affecting a single allele in *BRCA2* increase susceptibility to BC and OC, while certain bi-allelic mutations cause Fanconi anemia (FA) [49], in similar cases, it is crucial to establish whether the two mutations are in *cis* or in *trans* and, therefore, whether are on a same allele or on different alleles. To date, these two variants have never been reported in *trans* in literature. Furthermore, the *BRCA2* c.7008_2A>T (IVS13-2A>T) mutation, is unlikely to contribute to cancer risk in the context of the allele described here, since it lies downstream of another mutation that completely abolishes the synthesis of a functional gene product. These variants were confirmed to occur on the same chromosome (in *cis* phase) by retro-transcription RT-PCR analysis or segregation analysis in family studies [37,39,50].

We assume that these pathogenic variants are in *cis* because all tested family members show DM, none has inherited a single variant. This finding indicates that these pathogenic variants co-segregate thus establishing that they affect the same allele. Moreover, in carriers of these pathogenic variants, no traces of FA-associated tumors are present.

The *BRCA2* DM c.631G>A (p.Val211Ile)/c.7008-2A>T (IVS13-2AT) occur more frequently in Italian regions [36,37,38,39]. This allelic frequency in Italian regions supports the assumption that such pathogenic variants might be evident in geographically restricted areas and descend from a common ancestor, as suggested by the presence of a unique *BRCA2* allele in Italy.

The increased risk of cancer in DH carriers, as in patients with a single mutation in *BRCA* genes, is not limited to breast and ovarian cancer but also involves other cancers [34]. In line with this evidence, among the tumors reported in the analyzed DH *BRCA1/2* families, the most frequent was OC (21.4%), followed by BC, prostate cancer (PC), and CC, with a percentage of 14.3%, and bBC, breast and ovarian cancer (BOC), bladder cancer (BLC), leukemia (LEU), and laryngeal cancer (LAC), with a percentage of 7.1% (Figure 6). In a recent study, *BRCA1/2* mutation carriers displayed an increased risk for PC (3.4-fold increased risk in *BRCA1*, 8.6-fold increased risk in *BRCA2*) [51]. Moreover, the *BRCA2* mutations carriers have greater risk of bile duct, gall bladder, pancreatic, gastro-intestinal tumors, and melanoma [52], while the *BRCA1* mutations carriers of CC [53].

About the age of cancer development in the DH carriers, the occurrence of the first cancer should be earlier than in the carriers of a single *BRCA* mutation [7]. However, data about the younger age of DH carriers in BC development are conflicting. As described above, data concerning the *BRCA* DH incidence in non-Ashkenazi and in Italian populations are few, and often they have documented single patients rather than family studies. Claus reported DH in a female affected with BC and diagnosed at an early age of 37 years [24]. Musolino described DH in a female with BC diagnosed at 37 years [32]. Concolino reported DH in a female with bBC and age at diagnosis of 41 and 54 years [36]. Palmirotta reported that the median age at diagnosis for BC was 38 years, with a range of 26–76 [7]. In our patients, the mean age at BC diagnosis was 54.5 years (Table 5) but the range was 32–70 years, in line with data reported by Palmirotta.

Among the tumors reported in DM *BRCA2* families, the most frequent was BC, with a percentage of 76.2%, followed by bBC, male breast cancer (MBC), CC, pancreatic cancer (PAC), and LEU with a percentage of 4.8% (Figure 6). The tumors recurring in the families with DM are the same as in the carriers of *BRCA2* single mutations. Particularly, *BRCA2* mutations are found in up to 2% of PAC cases [54]. The *BRCA2* DM c.631G>A (p.Val211Ile)/c.7008-2A>T (IVS13-2A>T) was previously reported in a patient with PAC [40]. Accordingly, in family 5 with DM a case of PAC was reported, but the genetic test could not be assessed since the patient died. This data supports the hypothesis of a correlation between *BRCA2* and PAC. In relation to the age of cancer onset, in DM patient with PAC we did not observe a younger age compared with carriers of a single *BRCA2* mutation [55].

Overall, the literature data indicate that DH, although rare, is more frequently reported than DM (Table 6 and Table 7). Otherwise, our analysis shows a higher frequency of DM (0.5%) than DH (0.3%), in accordance with the data of Concolino, reporting a highest DM frequency (1.6%) compared to DH (0.8%) [36]. This different result could be due to the small number of samples tested in these two studies.

Analyzing the types of tumors occurring in families with DH and DM (see Figure 6), we observed OC and BOC only in families with DH but not with DM. This finding could be due to the presence of *BRCA1* mutations in DH cases; indeed, it is recognized that these mutations highly predispose to the OC onset. Likewise, an increased risk of OC in patients with *BRCA1/2* DH compared to *BRCA2* mutation carriers was found [8]. Furthermore, MBC was present in families with DM, whereas no cases were diagnosed in DH families, thus confirming that *BRCA2* mutations have a greater penetrance BC development in men [56]. Similarly, PAC was reported in families with DM but not with DH, further supporting the notion that *BRCA2* mutations are associated with an increased risk of PAC [57].

About the age of onset of various cancer types, we did not observe significant differences between families with DH and DM. The only discrepancy concerns CC (Table 5), which could have an earlier onset in families with DH, probably due to the presence of *BRCA1* in DH; indeed, a greater risk of CC at the age of about 50 years in *BRCA1* mutation carriers was reported [58].

In addition to *BRCA* genes, DH can also involve other cancer susceptibility genes. To date, we have shown patients with DH in *BRCA2*/*MSH2*, *BRCA2*/*APC*, *BRCA1*/*MSH6*, *BRCA1*/*MLH1*, *BRCA1*/*ATM*, *BRCA1*/*CHEK2*, *BRCA1*/*BLM*, and *BRCA1*/*APC* [59,60,61,62,63].

## 5. Conclusions

The detection of DH could have important clinical implications for the management of patients and for risk assessment in the family members of mutated patients. In order to identify all possible cases of DH in genes of high and moderate penetrance for cancer, a genetic test based on multiple genes involved in tumor predisposition syndromes is essential.

## Figures and Tables

**Table 1:** Probands with double heterozygosity (DH) in the *BRCA1/2* genes.

Proband Number	Gene	Pathogenic Variant	Molecular Consequence	dbSNP ID
1	*BRCA1* *BRCA2*	c.547+2T>A (IVS8+2T>A) c.2830A>T (p.Lys944Ter)	Splice Nonsense	rs80358047 rs80358533
2	*BRCA1* *BRCA2*	c.3752_3755GTCT (p.Ser1253fs) c.425+2T>C (IVS4+2T>C)	Frameshift Splice	rs80357868 rs876661045

**Table 2:** Probands with double mutation (DM) in the *BRCA2* genes.

Proband Number	Gene	Pathogenic Variant	Molecular Consequence	*dbSNP ID*
3	*BRCA2* *BRCA2*	c.631G>A (p.Val211Ile) c.7008-2A>T (IVS13-2A>T)	Splice Splice	rs80358871 rs81002823
4	*BRCA2* *BRCA2*	c.631G>A (p.Val211Ile) c.7008-2A>T (IVS13-2A>T)	Splice Splice	rs80358871 rs81002823
5	*BRCA2* *BRCA2*	c.631G>A (p.Val211Ile) c.7008-2A>T (IVS13-2A>T)	Splice Splice	rs80358871 rs81002823

**Table 3:** Families tested for DH in *BRCA1/2.*

Family Number	Family Members	Years	Diagnosis	Age at Onset	Genetic Test Result
1	proband father	49 78	bBC healthy	32	DH DH
	mother	68	healthy		NEG
	paternal aunt	74	healthy		NEG
	sister	48	healthy		NEG
	cousin female	52	BC-OC	39–46	DH
	cousin male	50	healthy		NEG
	nephew	27	healthy		DH
	nephew	21	healthy		DH
	niece	25	healthy		DH
2	proband mother	36 70	OC healthy	36	DH DH
	cousin male	40	CC	37	DH

bBC = bilateral breast cancer; OC = ovarian cancer; BC = breast cancer; CC: colon cancer.

**Table 4:** Families tested for DM in *BRCA2.*

Family Number	Family Members	Years	Diagnosis	Age at Onset	Genetic Test Result
3	proband mother	46 73	BC BC	45 69	DM DM
	cousin female	45	BC	43	DM
4	proband daughter	77 52	BC healthy	65	DM DM
	son	54	healthy		NEG
	grandchildren	27	healthy		NEG
	grandchildren	18	healthy		DM
	niece	55	BC	53	DM
	niece	43	BC	40	DM
	niece	56	BC	55	DM
	niece	47	BC	44	DM
	grand-niece	22	healthy		NEG
5	proband brother	62 59	BC healthy	60	DM NEG
	sister	56	bBC	43	DM
	daughter	35	healthy		DM
	daughter	41	healthy		NEG
	niece	35	healthy		NEG
	niece	30	healthy		NEG
	niece	33 †	BC	32	DM
	cousin female	70	BC	60	DM

bBC = bilateral breast cancer; BC = breast cancer; †: dead.

**Table 5:** Number and onset age of various type of cancers that occur in families with DH and DM.

	BC	bBC	OC	BOC	MBC	PC	LAC	CC	PAC	LEU	BLC
DH	2 (54.5)	1 (32)	3 (49)	1 (35)	-	2 (69)	1 (70)	2 (47.5)	-	1 (28)	1 (52)
DM	16 (50.6)	1 (43)	-	-	1 (73)	-	-	1 (78)	1 (63)	1 (36)	-

BC: breast cancer; bBC: bilateral breast cancer; OC: ovarian cancer; BOC: breast ovarian cancer; MBC: male breast cancer; PC: prostate cancer; LAC: laryngeal cancer; CC: colon cancer; PAC: pancreatic cancer; LEU: leukemia; BLC: bladder cancer.

**Table 6:** *BRCA1* and *BRCA2* DH reported worldwide (ClinVar nomenclature).

*BRCA1*	*BRCA2*	Cancer	Age at Diagnosis	Geographic Area	References
c.68_69delAG (185delAG) (p.Glu23fs)	c.5946delT (6174delT) (p.Ser1982Argfs*22)	BC OC BOC Healthy	35 at 57	Hungarian, Jewish, Ashkenazi J, Dutch, Israel	[9,10,11,12,13,14]
c.5266dupC (5382insC) (p.Gln1756Profs)	c.5946delT (6174delT) (p.Ser1982Argfs*22)	BC BOC	33 at 47	Ashkenazi J, Dutch	[11,13,15,16]
c.3768_3769AG (p.Glu1257fs)	c.5946delT (6174delT) (p.Ser1982Argfs*22)	BC BOC	30 at 41	Ashkenazi J, Australian	[17,18]
c.3695_3699GTAAA (p.Val1234fs)	c.1813dupA (p.Ile605Asnfs)	BC OC/CC	40,61	Germany	[19]
c.2389G>T (p.Glu797Ter)	c.3068dupA (p.Asn1023Lysfs)	BC	35	Scottish	[20]
c.5080G>T (p.Glu1694Ter)	c.6405_6409del (p.Asn2135fs)	OC	49	German	[21]
c.5123C>A (p.Ala1708Glu)	c.6275_6276del (p.Leu2092fs)	BC	28	Spanish	[22]
c.1504_1508del (p.Leu502fs)	c.2796_2797CA (p.Thr933fs)	BC	26	Korea	[23]
c.4981G>T (p.Glu1661Ter)	c.5946_5949del (p.Ser1982fs)	BC	33	Korea	[23]
c.2685_2686del (p.Pro897fs)	c.3487del (p.Asp1163fs)	BOC	40,45	Dutch	[13]
c.2685_2686del (p.Pro897fs)	c.4449del (p.Asp1484fs)	BC	50	Dutch	[13]
c.962G>A (p.Trp321Ter)	c.3170_3174del (p.Lys1057fs)	BC	37	Mixed European	[24]
c.4287C>A (p.Tyr1429Ter)	c.7738C>T (p.Gln2580Ter)	BC	37	Italy	[32]
c.3331_3334del (p.Gln1111fs)	c.631+2T>G (IVS7+2T>G)	BC CC	34,35	Australian	[25]
c.5266dupC (5382insC) (p.Gln1756Profs)	c.5796_5797del (p.His1932fs)	BC/OC	44	Italy	[33]
c.5096G>A (p.Arg1699Gln)	c.631+4A>G (IVS7+4A>G)	BOC	53,59	Danish	[26]
c.835_835delC (p.His279Metfs)	c.8195T>G (p.Leu2732Ter)	BC	43	Northern Italy	[34]
c.1687C>T (p.Gln563Ter))	c.6697C>T (p.Gln2157Ter)	BC	30	Northern Italy	[34]
c.2401_2402TG (p.Val802fs)	c.4284dup (p.Gln1429fs)	BC	46	Northern Italy	[34]
c.3916_3917del (p.Leu1306fs)	5608delG (p.Val1794Ter)	BC	52	Northern Italy	[34]
c.1961del (p.Lys654fs)	c.1672del (p.Ile558fs)	OC	50	Non-Ashkenazi	[27]
c.5266dupC (5382insC) (p.Gln1756Profs)	c.4827_4828TG (p.Val1610fs)	BC	40	Ashkenazi	[27]
c.5266dupC (5382insC) (p.Gln1756Profs)	c.5645C>A (p.Ser1882Ter)	BC	37	Germany	[19]
c.68_69delAG (185delAG) (p.Glu23fs)	c.5718_5719delCT (p.Leu1908Argfs)	BC	32	Germany	[19]
c.962G>A (p.Trp321Ter)	c.2231C>G (p.Ser744Ter)	BC	31	Germany	[19]
c.3910delG (p.Glu1304Lysfs)	c.2830A>T (p.Lys944Ter)	BC	40	Germany	[19]
c.5277+1de (IVS20+1delG)	c.658_659del (p.Val220fs)	BC	39	Germany	[19]
c.390C>A (p.Tyr130Ter)	c.3018del (p.Gly1007fs)	BC	26,45	Germany	[28]
c.4981G>T (p.Glu1661Ter)	c.5946_5949del (p.Ser1982fs)	BC	33	Germany	[28]
c.3627dupA (p.Glu1210Argfs)	c.6724_6725delGA (p.Asp2242Phefs)	BC	26	Germany	[28]
c.5030_5033del (p.Thr1677fs)	c.1399A>T (p.Lys467Ter)	BC	35	Germany	[28]
c.2641G>T (p.Glu881Ter)	c.8162T>A (p.Leu2721His)	BC	42	South African	[29]
c.547+2T>A (IVS8+2T>A)	c.2830A>T (p.Lys944Ter)	bBC	32	Southern Italy	[35]
c.188T>A (p.Leu63Ter)	c.5576_5579del (p.Ile1859fs)	BC	59	Japan	[30]
c.1687C>T (p.Gln563Ter)	c.9976A>T (p.Lys3326Ter)	BC	40	Southern Italy	[7]
c.5095C>T (p.Arg1699Trp)	c.1238delT (p.Leu413HisfsTer17)	bBC	41,54	Italy	[36]
c.547+2T>A (IVS8+2T>A)	c.2830A>T (p.Lys944Ter)	BC	32	Southern Italy	This paper
c.3752_3755delGTCT (p.Ser1253fs)	c.425+2T>C (IVS4+2T>C)	OC	36	Southern Italy	This paper

**Table 7:** *BRCA2* DM reported worldwide (ClinVar nomenclature).

*BRCA2*	Cancer	Age at Diagnosis	Geographic Area	Reference
c.1547del (p.Phe516fs) and c.4599A>C (p.Lys1533Asn)	LEU	37	Korea	[23]
c.2905C>T (p.Gln969Ter) and c.6447_6448dupTA (p.Lys2150IlefsTer19)	BC	49	Italy	[36]
c.631G>A (p.Val211Ile) and c.7008-2A>T (IVS13-2A>T)	BOC	45 at 65	Italy USA	[36,37,38,39] This paper [40]
c.4889C>G (p.Ser1630Ter) and c.5344C>A (p.Gln1782Lys)	BC	46	White	[24]
